# Longistatin, a Plasminogen Activator, Is Key to the Availability of Blood-Meals for Ixodid Ticks

**DOI:** 10.1371/journal.ppat.1001312

**Published:** 2011-03-10

**Authors:** Anisuzzaman, M. Khyrul Islam, M. Abdul Alim, Takeharu Miyoshi, Takeshi Hatta, Kayoko Yamaji, Yasunobu Matsumoto, Kozo Fujisaki, Naotoshi Tsuji

**Affiliations:** 1 Department of Global Agricultural Sciences, Graduate School of Agricultural and Life Sciences, The University of Tokyo, Tokyo, Japan; 2 National Institute of Animal Health, National Agricultural and Food Research Organization, Tsukuba, Japan; 3 Animal Functional Genomics Laboratory, Department of Primary Industries, Attwood, Victoria, Australia; 4 National Research Centre for Protozoan Diseases, Obihiro University of Agriculture and Veterinary Medicine, Obihiro, Japan; 5 School of Frontier Veterinary Medicine, Kagoshima University, Kagoshima, Japan; Institut Pasteur, France

## Abstract

Ixodid ticks are notorious blood-sucking ectoparasites and are completely dependent on blood-meals from hosts. In addition to the direct severe effects on health and productivity, ixodid ticks transmit various deadly diseases to humans and animals. Unlike rapidly feeding vessel-feeder hematophagous insects, the hard ticks feed on hosts for a long time (5−10 days or more), making a large blood pool beneath the skin. Tick's salivary glands produce a vast array of bio-molecules that modulate their complex and persistent feeding processes. However, the specific molecule that functions in the development and maintenance of a blood pool is yet to be identified. Recently, we have reported on longistatin, a 17.8-kDa protein with two functional EF-hand Ca^++^-binding domains, from the salivary glands of the disease vector, *Haemaphysalis longicornis*, that has been shown to be linked to blood-feeding processes. Here, we show that longistatin plays vital roles in the formation of a blood pool and in the acquisition of blood-meals. Data clearly revealed that post-transcriptional silencing of the longistatin-specific gene disrupted ticks' unique ability to create a blood pool, and they consequently failed to feed and replete on blood-meals from hosts. Longistatin completely hydrolyzed α, β and γ chains of fibrinogen and delayed fibrin clot formation. Longistatin was able to bind with fibrin meshwork, and activated fibrin clot-bound plasminogen into its active form plasmin, as comparable to that of tissue-type plasminogen activator (t-PA), and induced lysis of fibrin clot and platelet-rich thrombi. Plasminogen activation potentiality of longistatin was increased up to 4 times by soluble fibrin. Taken together, our results suggest that longistatin may exert potent functions both as a plasminogen activator and as an anticoagulant in the complex scenario of blood pool formation; the latter is critical to the feeding success and survival of ixodid ticks.

## Author Summary

Ixodid ticks are serious blood-sucking ectoparasites that are essentially dependent on blood-meals from hosts for survival. The feeding mechanism of hard ticks, however, is very complex and is quite different from that of blood-sucking insects that suck blood rapidly and directly from blood vessels. Hard ticks suck blood for quite a long period (5−10 days or more) by making a large blood pool beneath the skin. Despite the fact that mammalian hosts are armored with strong blood clotting machineries, ticks manage to keep the blood in a fluid state and to maintain a blood pool until a full blood-meal is secured. However, very little is known about the anti-hemostatic mechanisms by which ticks cleverly manipulate the host's blood clotting cascade and make blood-meals available. Here, we show that longistatin, a salivary gland protein identified from the tick *Haemaphysalis longicornis*, can efficiently manipulate the host's blood clotting machineries, such as fibrinogen, fibrin and plasminogen, and can help prevent cascade of blood clotting. In conclusion, longistatin plays a crucial role in the blood-feeding success of hard ticks and can be a novel therapeutic target against ticks and tick-borne diseases, including human occlusive cardiovascular diseases like thrombosis.

## Introduction

Blood coagulation is a very complex but well-synchronized biochemical process by which blood forms a clot and the damaged blood vessel is sealed by a platelet-rich fibrin plug leading to hemostasis. Any damage to the vascular beds due to laceration of tissues exposes tissue factor from the endothelium to the circulating blood, which initiates coagulation cascades. Once coagulation is initiated, the process leads to the generation of thrombin, which converts fibrinogen to fibrin, the building block of a hemostatic plug [1]–[5]. In contrast, fibrinolysis is an enzymatic process wherein a fibrin clot is dissolved. Normally, in the body, both coagulation and fibrinolytic processes are precisely regulated by the measured participation of zymogens, activators, inhibitors, cofactors and receptors. Plasmin is the major fibrinolytic enzyme and is derived from the limited proteolytic cleavage of plasminogen, the circulating plasma zymogen, by its physiological activators such as tissue-type plasminogen activator (t-PA) and urokinase-type plasminogen activator (u-PA) [6]–[9]. Homeostasis in blood fluidity is not only vital for humans but also for hematophagous animals, which have to counteract their hosts' hemostatic mechanisms and/or facilitate the fibrinolytic process to keep the blood in a fluid state during acquisition and digestion of blood-meals. Therefore, it is believed that blood sucking-animals require an extensive spectrum of anticoagulant and/or fibrinolytic mechanisms to maximize their feeding as part of their diverse survival strategies. Thus, hematophagous animals are thought to possess anticoagulant and/or fibrinolytic proteins that have been acquired during their evolution [1], [10], [11].

The ixodid ticks (Arthropoda: Ixodidae), popularly known as hard ticks, are notorious ectoparasites and live entirely on nutrients derived from host's blood protein, hemoglobin. Blood-feeding is an essential biologic phenomenon for the survival of hard ticks [12]–[14]. These ectoparasites firmly attach to hosts using their deeply penetrating mouthparts on hosts' skin and cause dermatitis and severe anemia. In addition to the direct severe adverse effects on health and productivity, ticks serve as a unique vector of various deadly diseases, such as lyme disease, tick-borne encephalitis, Rocky Mountain spotted fever, babesiosis, theileriosis and anaplasmosis during hematophagy. Ticks are only second to mosquitoes as vectors of diseases of humans and animals [15]–[20]. Unlike rapidly feeding hematophagous insects that suck blood directly from the blood vessels within a couple of seconds, the hard ticks feed on blood-meals for a long time (5−10 days or more), making a large blood pool beneath the host's skin. Although size of the blood pool produced by different species of ticks varies but it is an essential feature in the feeding mechanism of ticks [13], [21]. Blood pools contain copious unclotted blood and exudates, especially in the rapid phase of feeding [1], [21]. It is of current interest to look at the molecular scenario inside the blood pool that maintains a large volume of unclotted blood underneath the skin. Prior studies suggest that ixodid ticks, the smart pharmacologists, produce a vast array of pharmacologically active bio-molecules that are injected into the feeding lesions during persistent blood-feeding processes, and play crucial modulatory roles in their feeding success, especially to keep the blood in a fluid state in the blood pool and within the gut as well [22]–[26]. However, the precise molecular mechanism(s) that prevents blood coagulation and initiates fibrinolysis in the blood pool to facilitate successful acquisition of blood-meals is still unclear. Previously, we have shown that longistatin, an ixodid tick salivary gland-derived bioactive molecule, is functionally linked to the blood-feeding processes. The molecule was shown to be secreted into the blood pool created by ticks while they attach on a robust mammalian host and suck blood to engorge [27].

Here, we demonstrate that longistatin-specific gene-knockdown ixodid ticks such as *Haemaphysalis longicornis* completely lost their unique ability to create a blood pool and consequently were unable to feed and engorge on blood-meals as verified by RNA interference (RNAi) tool. Furthermore, we describe that longistatin binds with fibrin and specifically catalyzes the activation of plasminogen into plasmin and hydrolyzes fibrinogen *in vitro*; the latter is known to be a major component of cross-linked fibrin polymer. To the best of our knowledge, longistatin is the first plasminogen activator, isolated and characterized from hematophagous arthropods.

## Results

### Injection of ds*longistatin* inhibits the transcription and translation of endogenous longistatin

**Figure 1:**
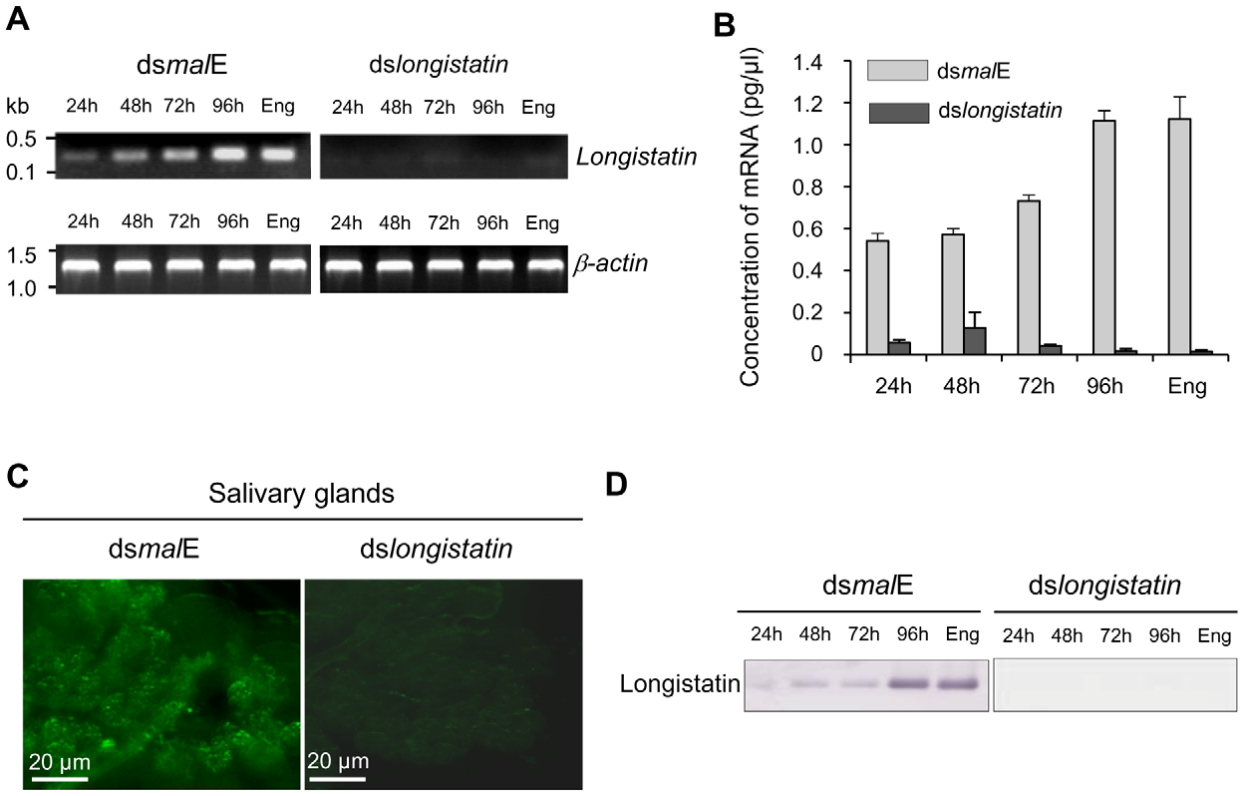
Post-transcriptional silencing of longistatin-specific gene in adult ticks by injecting dsRNA. (A) Semiquantitative RT-PCR analysis. One microgram of *longistatin* dsRNA was injected into the hemolymph of ticks of the RNAi-treated group. Ticks of the control group were treated with 1 µg of *mal*E dsRNA. Actin was used as an internal control. Eng, engorged. (B) Quantitative RT-PCR using total RNA and primers specific for longistatin as in A. Eng, engorged. (C) *In situ* detection of longistatin expression in ticks' salivary glands. Salivary glands from the ticks of control and RNAi-treated groups. Endogenous longistatin was reacted with mouse anti-longistatin sera (1∶100). (D) Effect of gene silencing on longistatin post-translation by Western blot analysis. Salivary gland extracts were electrophoresed and transferred onto nitrocellulose membrane. Endogenous longistatin was probed with mouse anti-longistatin (1: 100). Eng, engorged.

Total RNA isolated from salivary glands of ticks of different feeding phases of both control and treated groups was analyzed by reverse transcription-PCR (RT-PCR) and quantitative RT-PCR (qRT-PCR) to demonstrate the effect of RNAi on the expression of longistatin-specific mRNA. The RT-PCR data revealed that injection of ds*longistatin* completely abolished the detectable mRNA expression corresponding to the longistatin-specific gene in ticks (Figure 1A). Our qRT-PCR data also supported this finding. However, in the RNAi-treated group, longistatin-specific transcript, although very low compared with that of control, was detected in ticks at 24, 48 and 72 h of feeding only by qRT-PCR (Figure 1B). This variation in the detection of longistatin-specific mRNA by RT-PCR and qRT-PCR may be due to the sensitivity of the techniques. Furthermore, the presence of a detectable level of mRNA in the RNAi-treated group of ticks might be due to individual variations in the tick population. Here, we used pools of salivary-gland extracts, isolated from three randomly selected ticks in each feeding phase; thus, it is quite reasonable that the effects of RNAi were not exactly uniform in each and every microinjected tick. Longistatin-specific gene expression was detected in its normal pattern [27] in ticks injected with dsRNA complementary to the gene encoding maltose-binding protein in *Escherichia coli* (ds*mal*E) (Figures 1A and B), indicating that injection of ds*longistatin* caused disruption of longistatin-specific mRNA transcription. For *in situ* detection of the effect of RNAi on the translation of endogenous longistatin, salivary glands were collected from partially fed (96 h) ticks of both treated and control groups and were subjected to immunofluorescence staining using mouse anti-longistatin sera. Longistatin-specific reactions were almost absent in ds*longistatin*-injected ticks whereas binding of mouse anti-longistatin antibody was detected in the salivary glands of ds*mal*E-injected ticks (Figure 1C), suggesting that injection of ds*longistatin* efficiently silenced longistatin-specific mRNA expression and subsequent translation of longistatin. To further validate our results regarding *in vivo longistatin* gene silencing, we conducted Western blot analysis using salivary gland extracts collected from both treated and control ticks. Longistatin-specific bands were detected only in the salivary gland extracts of ds*mal*E-injected ticks and longistatin was upregulated with the feeding process of ticks (Figure 1D). These findings further reinforced the evidence of longistatin-specific gene silencing by ds*longistatin* injection in ticks.

### RNAi-treated ticks failed to develop a blood pool and were unable to feed blood-meals from hosts

**Figure 2:**
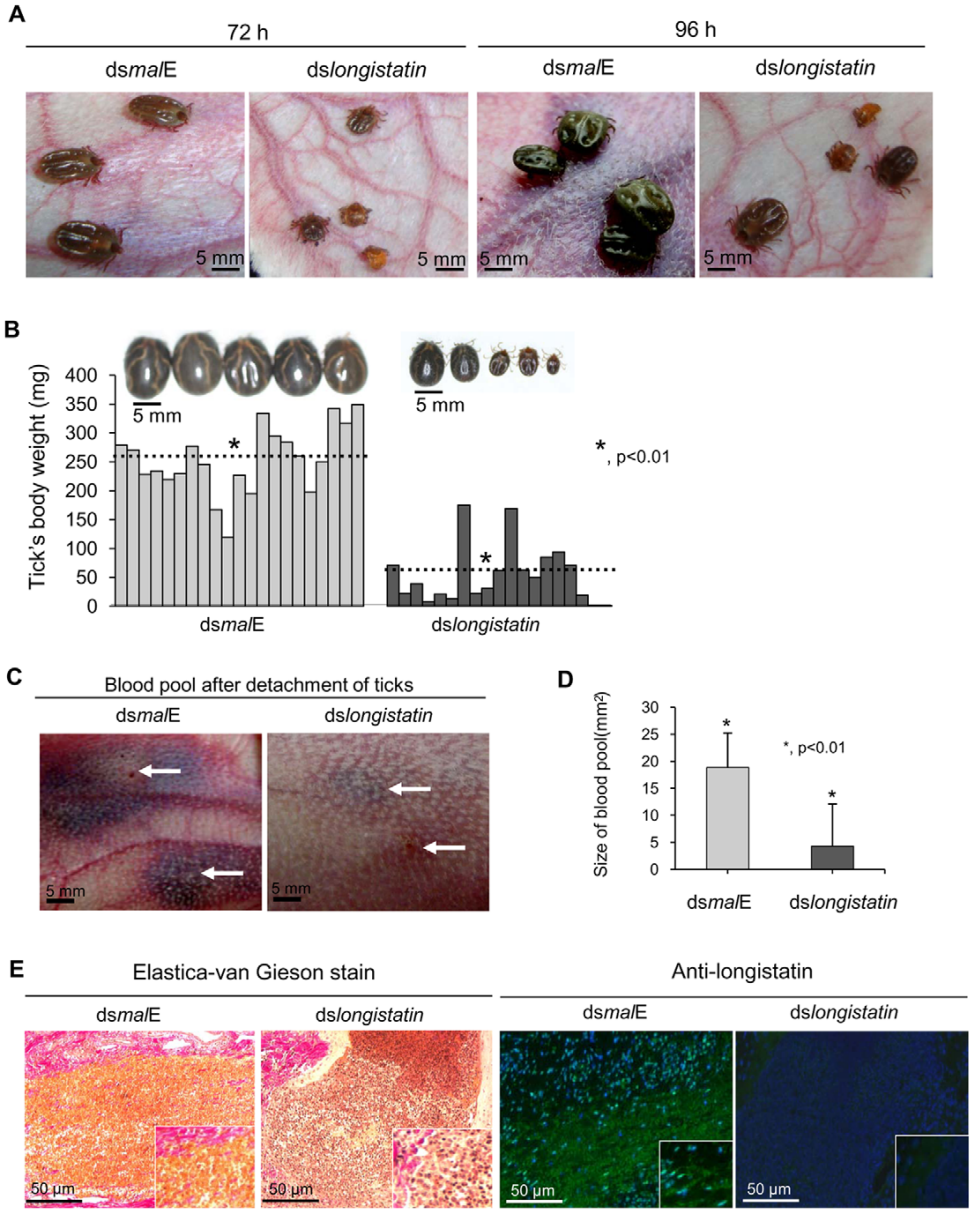
Effects of post-transcriptional silencing of *longistatin* gene on blood pool formation and blood feeding. (A) Impact of longistatin-specific mRNA silencing on blood-meal feeding from hosts. Adult ticks were injected with *longistatin* dsRNA or *mal*E dsRNA (1 µg/tick) and were allowed to feed on a tick-naïve rabbit. RNAi-treated ticks failed to replete. (B) Postengorgement weight was significantly (p<0.01) lower in the RNAi-treated group than that of the control group. Dotted lines indicate mean±SD of body weight of ticks. (C) Effects of post-transcriptional silencing of *longistatin* gene on blood pool formation. RNAi was performed and ticks were fed in the same manner as in A. RNAi-treated ticks failed to establish a prominent blood pool. Arrows indicate site of tick attachment. (D) Blood pools were significantly (p<0.01) smaller in the RNAi-treated group. (E) Histopathological changes were studied using EVGS. Longistatin was detected in the feeding lesions of ticks on the host's tissues using mouse anti-longistatin sera (1∶100).

All ticks microinjected with dsRNA were active and healthy during the incubation period. After placement on rabbits' ears, all ticks of both treated and control groups actively attached. However, in the ds*longistatin*-injected group, 3 (2.5%) ticks were found dead at 72 h of attachment. All ticks in the ds*mal*E-injected group reached to repletion and detached by day 6 post-attachment. Notably, ds*longistatin* injection was shown to hamper the feeding of ticks. These ticks were poorly fed and most of them failed to engorge (Figure 2A). Only two ticks (1.66%) dropped off the host following engorgement in the RNAi group. The mean body weight of the ticks collected after 6 days of feeding was significantly (P<0.01) lower in the RNAi-treated group (53.53±50.38 mg) than that of the control group (253.43±57.91 mg) (Figure 2B). Visible phenotypic changes were also obvious among the ticks of the treated and control groups. Ticks of the treated group, despite 6 days of feeding, were very small with a dull cuticle and devoid of normal cuticular wrinkling. In contrast, ticks of the control group, which dropped off the host following full engorgement, were large and glossy in appearance with dorsal cuticular wrinkling (Figure 2B).

A marked difference between blood pools induced by the ticks of RNAi-treated and control groups was observed. Large blood pools were developed at the site of attachment of each tick of the control group. Grossly, blood pools were large enough with an estimated mean size of 19.53±7.85 mm^2^, containing a considerable amount of exudates and blood. The affected area was cyanotic or dark bluish in color. Blood vessels in the vicinity of the blood pool were congested and distended. The surrounding area of the blood pool was markedly hyperemic. On the contrary, most of the ticks of the RNAi-treated group failed to establish a feeding lesion. Importantly, significantly (p<0.01) smaller blood pools (4.25±6.38 mm^2^) were detected in few individual ticks. Also, no prominent gross pathological changes were detected at the site of attachment of RNAi-treated ticks (Figures 2C and D). To study the histological features, we stained the thin tissue sections of the blood pool areas with Elastica-van Gieson stain (EVGS) where hemorrhagic areas exhibited a bright golden yellow color, indicating that blood pools, developed at the site of attachment of the ds*mal*E-injected ticks, were flooded with RBC. Notably, hemorrhagic changes were not detected at the biting areas of ticks of the RNAi-treated group (Figure 2E), suggesting that the *longistatin* gene plays vital roles in the formation and maintenance of a blood pool as preceded by marked hemorrhage into tick-feeding lesions. In addition, an attempt was made to detect endogenous longistatin in the feeding lesions produced by the ticks of both groups using mouse anti-longistatin sera. Longistatin-specific bright green fluorescence was detected at the well-developed blood pool areas produced by the ds*mal*E-injected ticks but no such reaction was detected at the feeding lesions induced by ds*longistatin*-injected ticks (Figure 2E), which further suggests a possible role of longistatin in the blood pool formation.

### Longistatin degrades fibrinogen and delays fibrin clot formation

**Figure 3:**
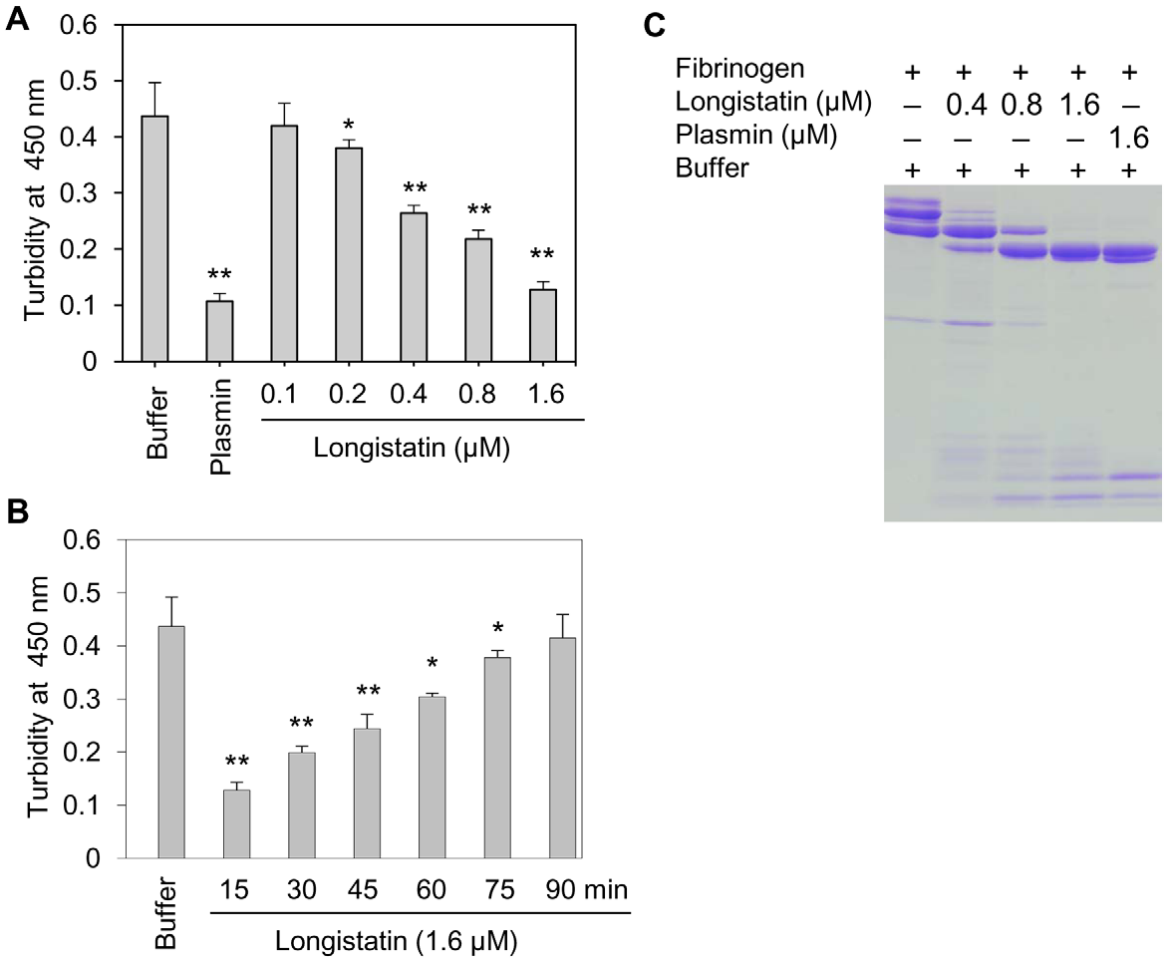
Anti-coagulation and fibrinogenolytic activity of longistatin. (A) Effects of longistatin on the formation of fibrin clot. Fibrinogen (7.5 mM) was pre-incubated in a buffer in the absence or presence of longistatin (0.1, 0.2, 0.4, 0.8 and 1.6 µM) or plasmin (1.6 µM) and then thrombin was added (0.10 NIH unit/µl) as described in Materials and Methods. Clot formation was detected visually and also by determining changes in turbidity at OD_450_ using a spectrophotometer after 15 min. (B) Longistatin (1.6 µM) delayed fibrin clot formation up to 90 min. Fibrinogen was incubated in the absence or presence of longistatin (1.6 µM) following the same procedures as mentioned in A and then thrombin was added. OD_450_ was measured at 15 min intervals. (C) Fibrinogenolytic effect of longistatin. Fibrinogen (7.5 mM) was incubated in the absence or presence of longistatin (0.4, 0.8 and 1.6 µM) or plasmin (1.6 µM). Samples were collected at the indicated time period and were subjected to 12.5% SDS–PAGE analysis under reducing conditions. A gradual degradation of the α, β and γ chains of fibrinogen was detectable with the concomitant deposition of degraded products. Asterisks (^*^) indicate that the difference compared with the negative control group (buffer only) is significant as determined by Student's *t*-test with unequal variance (^*^p<0.05, ^**^p<0.01).

To judge the anticoagulant function, longistatin was reacted with several coagulation factors (viz., thrombin, factors VIIa and Xa) using different approaches but none of them was affected by longistatin. Interestingly, longistatin was found to delay fibrin clot formation, degrade fibrinogen and efficiently activate plasminogen to its active form, plasmin. To evaluate the anticoagulation potential of longistatin, fibrinogen (7.5 mM) was pre-incubated in the absence or presence of longistatin (0.1, 0.2, 0.4, 0.8 and 1.6 µM) or plasmin (1.6 µM) and then thrombin (0.10 NIH unit/µl) was added as described in Materials and Methods. Longistatin significantly (p<0.01) interrupted the normal fibrin clot formation in a concentration-dependent manner. In the absence of longistatin, fibrin clot was formed within 15 min, but in the presence of longistatin (1.6 µM) or plasmin (1.6 µM), no visible clot was developed within this time (Figure 3A). Longistatin was shown to delay the formation of a visible fibrin clot. Fibrin clotting time was significantly extended (up to 90 min) at a concentration of 1.6 µM longistatin (Figure 3B), indicating potent anticoagulant activity of longistatin. To explore the fibrinogenolytic potential, fibrinogen was incubated in the absence or presence of longistatin (0.4, 0.8 and 1.6 µM) or plasmin (1.6 µM) and was subjected to SDS–PAGE analysis. Longistatin potently degraded the α, β and γ chains of fibrinogen in a concentration-dependent manner. Longistatin completely hydrolyzed all three chains of fibrinogen at 1.6 µM concentration, as it was done by 1.6 µM plasmin (Figure 3C), implying that longistatin is an efficient fibrinogenolytic protease of ixodid ticks.

### Longistatin activates plasminogen in the presence of soluble fibrin

**Figure 4:**
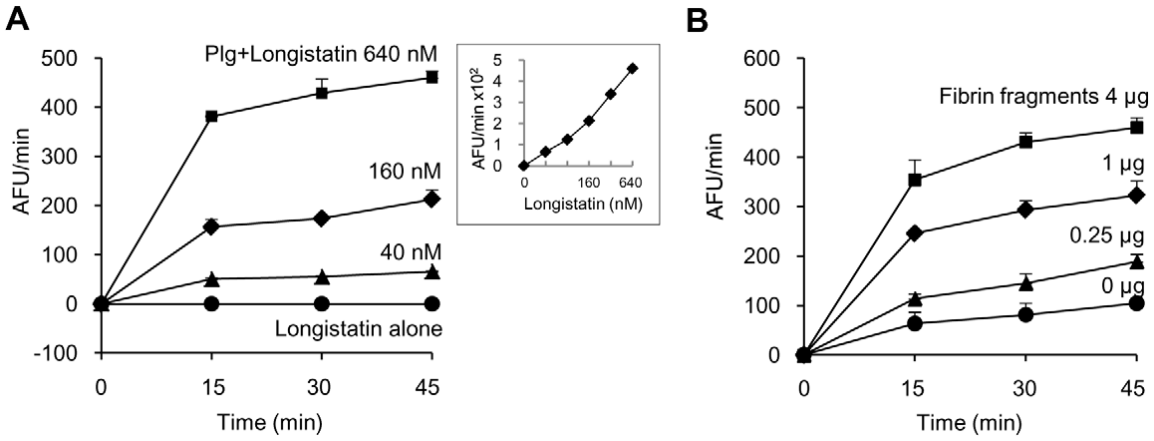
Plasminogen activation by longistatin. (A) Longistatin (40, 80, 160, 320 and 640 nM) was incubated without or with plasminogen (0.24 units) adding fibrin CNBr fragments (4 µg) in a total volume of 200 µl of buffer (50 mM Tris–HCl, pH 7.5; 100 mM NaCl and 5 mM CaCl_2_) at 25°C for 2 h. Then, plasmin-specific fluorogenic substrate (100 µM, final concentration) was added and substrate hydrolysis was monitored by measuring excitation and emission wavelengths of 360 nm and 460 nm, respectively, at 15 min intervals. *Inset*, initial rate of plasminogen activation at different concentrations of longistatin. (B) Effects of fibrin CNBr fragments on the activation of plasminogen by longistatin. Plasminogen (0.24 units) was incubated with longistatin (640 nM) in the absence or presence of fibrin CNBr fragments (0.25, 1 and 4 µg) as described in Materials and Methods. All assays were performed in triplicate.

Initially, we determined the plasminogen activation potential of longistatin by measuring the amidolytic activity of activated plasminogen on the plasmin-specific fluorogenic substrate. Data revealed that the initial rate of plasminogen activation was proportional to the concentration of longistatin. Hydrolysis of plasmin-specific substrate sharply increased with the increase of concentration of longistatin. However, longistatin alone, even at a higher concentration (640 nM), was not able to induce hydrolysis of plasmin-specific fluorogenic synthetic substrate (Figure 4A), indicating that longistatin activated plasminogen into its active form, plasmin, which hydrolyzed the synthetic substrate releasing MCA. Most interestingly, plasminogen activation potential of longistatin was significantly increased in the presence of soluble fibrin and induced robust amidolytic activity. Addition of fibrin cyanogen bromide (CNBr) fragments (4 µg) to the assay mixture caused an increase of plasminogen activation rate up to 4 times higher than that in the absence of fibrin CNBr fragments (Figure 4B). CNBr fragments of fibrin, the soluble fibrin, is usually regarded as a t-PA stimulator and increases the rate of plasminogen activation about 5 times [28], [29]. Plasminogen was sparingly affected by longistatin in the absence of soluble fibrin (Figure 4B), indicating the extraordinary specificity of longistatin towards fibrin-bound plasminogen rather than free circulating plasminogen.

### Longistatin is able to lyse fibrin clot by activating plasminogen

**Figure 5:**
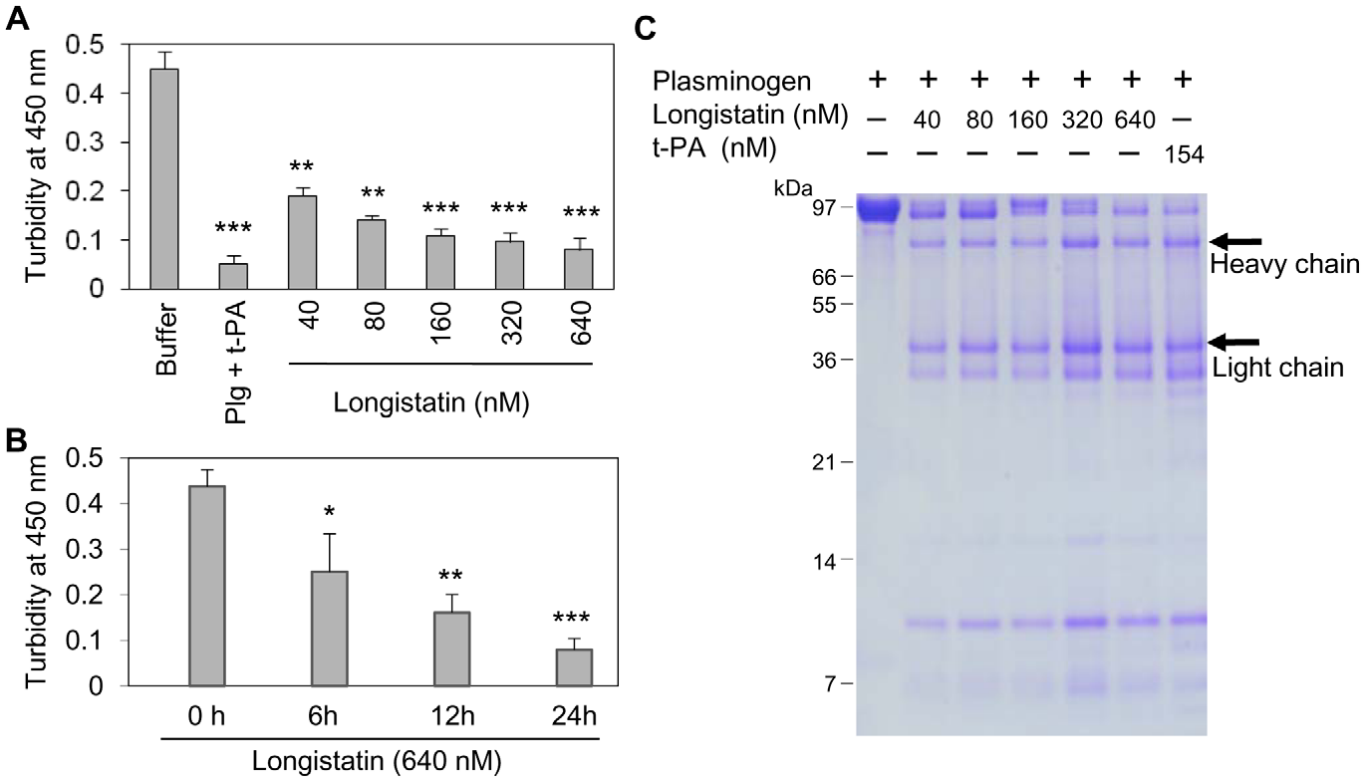
Longistatin induced fibrinolysis by activating plasminogen. (A) Fibrin clot was formed by incubating fibrinogen (7.5 mM) and thrombin (0.10 NIH unit/µl) and was incubated in the presence of plasminogen-t-PA/-longistatin (40, 80, 160, 320 and 640 nM) mixture or buffer only at 25°C for 24 h. Clot lysis was measured at OD_450_. Plasminogen induced complete lysis of fibrin clot in the presence of 640 nM longistatin or 154 nM t-PA. (B) Time-dependent activation of plasminogen by longistatin with concomitant lysis of fibrin clot. (C) Cleavage of plasminogen into the heavy and light chains. Digested product of fibrin clot was electrophoresed by 12.5% SDS–PAGE. Asterisks (^*^) indicate that the difference compared with the negative control group (buffer only) is significant as determined by Student's *t*-test with unequal variance (^*^p<0.05, ^**^p<0.01, ^***^p<0.001).

Although plasmin has enzymatic activity towards a broad spectrum of substrates, but the fibrin clot is considered as its main native substrate [30]. To further evaluate the plasminogen activation efficiency of longistatin, fibrin polymer was incubated with plasminogen–longistatin/-t-PA mixture or with buffer only at 25°C for 24 h. Data revealed that, in the presence of either longistatin or t-PA, plasminogen was able to induce lysis of fibrin clot. Visual observation and spectrometric analysis at 450 nm (OD_450_) revealed that the fibrinolytic potential of plasminogen, in the presence of longistatin, was directly proportional to the concentration of the recombinant protein used. These results firmly suggest that longistatin activates plasminogen to an active plasmin in a concentration-dependent manner, which in turn lyses the visible fibrin clot. Longistatin caused complete lysis of fibrin clot in the nanomolar range (Figures 5A and B). SDS–PAGE analysis showed that longistatin efficiently cleaved plasminogen into its heavy and light chains. These results are comparable to those of purified, active and commercially available t-PA (Figure 5C). Here, we also observed that longistatin activated a sufficient amount of plasminogen only in the presence of fibrin clot and induced profound fibrinolysis (data not shown).

### Longistatin binds with fibrin

**Figure 6:**
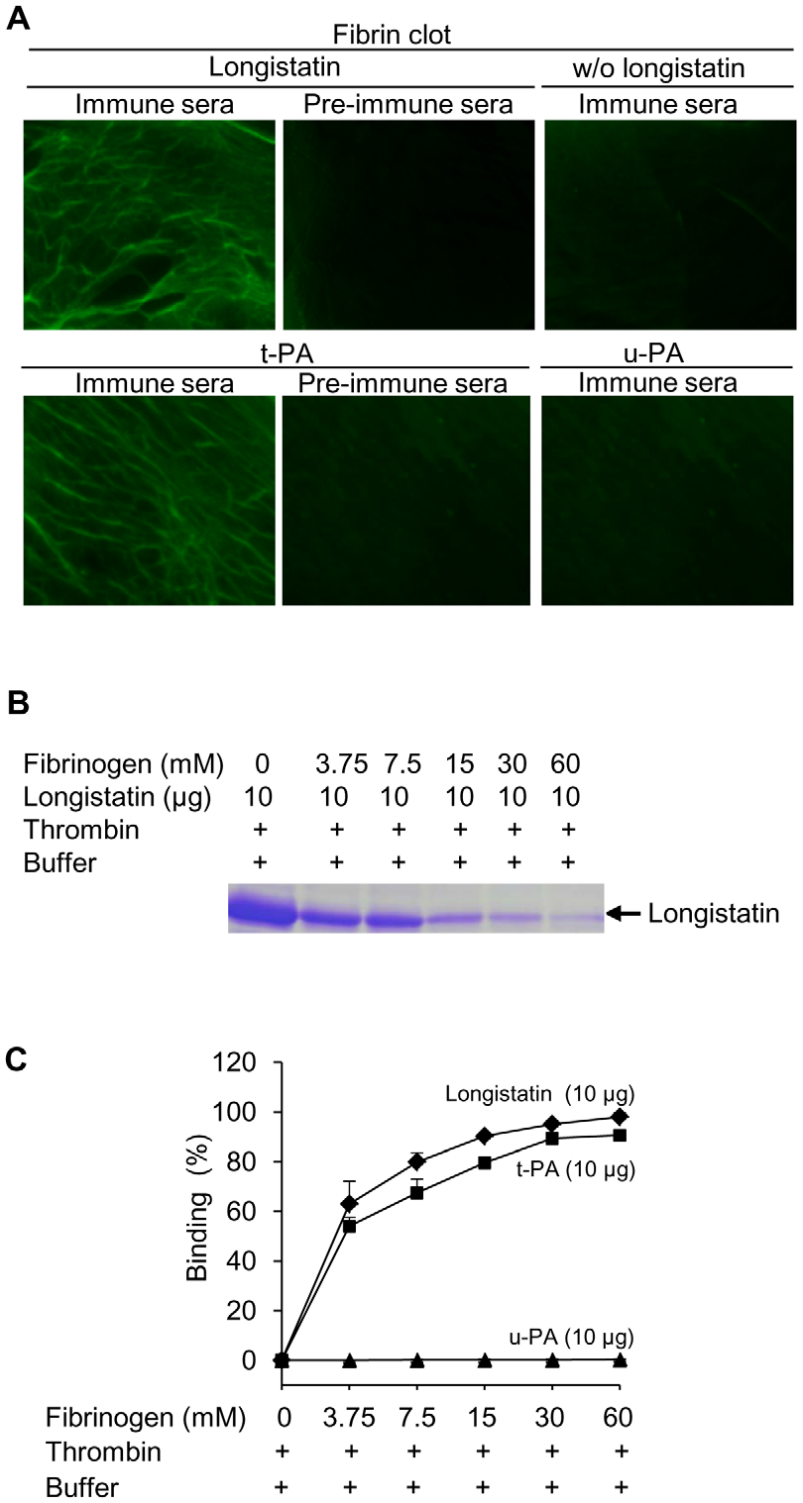
Binding of longistatin with fibrin clot. (A) Detection of longistatin bound on fibrin meshwork. Fibrinogen at different concentrations (3.75, 7.5, 15, 30 and 60 mM; final concentration) was mixed in the absence or presence of longistatin (10 µg) or an equal amount of t-PA or u-PA in a buffer (50 mM Tris–HCl, pH 7.5; 100 mM NaCl and 5 mM CaCl_2_) and thrombin (0.10 NIH unit/µl) was added immediately and was incubated at 25°C for 1 h. The clot was treated with anti-longistatin (1∶100), anti-t-PA (1∶100), anti-u-PA (1∶20) or pre-immune sera (1∶100). Bound antibodies were detected using green fluorescent-labeled secondary antibody (Alexa Flour 488 goat anti-mouse IgG). (B) Supernatant was analyzed by 12.5% SDS–PAGE under reducing conditions. (C) The target protein was extracted from the supernatant and its concentration was determined using micro-BCA reagent as described in Materials and Methods. The results are expressed as percentage of longistatin/t-PA/u-PA bound to the fibrin clot. Data represent mean ± SD, n = 3.

To explore the fibrin-binding capability of longistatin, we conducted fibrin-binding assays. Longistatin-specific green fluorescence was detected in the fibrin meshwork when purified recombinant longistatin was incorporated with fibrin and reacted with anti-longistatin sera. However, longistatin-specific fluorescent reaction was completely absent in the absence of longistatin in the reaction mixture or when longistatin-impregnated fibrin meshwork was treated with pre-immune sera (1∶100), indicating that longistatin is able to bind with fibrin clot. To compare the fibrin binding of longistatin, we used t-PA as a positive control because t-PA is known to bind with fibrin [31]. Here, t-PA-specific fluorescence was only detected when t-PA added fibrin was treated with anti-t-PA mouse monoclonal antibody (1∶100), but no such reaction was visible in the presence of pre-immune sera (normal serum) at the same concentration. To evaluate the specificity of the fibrin-binding assays, we used u-PA as a negative control because u-PA does not bind with fibrin [32]. u-PA-specific reaction was not detected in the fibrin meshwork even in the presence of a relatively high concentration (1∶20) of anti-u-PA antibody (Figure 6A). By using SDS−PAGE analysis, we also observed that the concentration of residual longistatin was markedly reduced after fibrin clot formation (Figure 6B), which reinforced the assertion that longistatin was bound with the fibrin clot. Furthermore, we determined the concentration of longistatin/t-PA in the supernatant obtained from the fibrin-binding assay mixer and data revealed that the percentage of binding of longistatin/t-PA with fibrin meshwork was directly proportional to the amount of fibrin. Longistatin binding was 97.93%±1.68% when 60 mM fibrinogen was used to produce fibrin clot and, under the same conditions, binding of t-PA was 90.6%±1.2%. Specificity of the binding was evidenced by the absence of u-PA binding (Figure 6C). To evaluate the fibrin-binding potentials of longistatin, we determined the fibrin-binding parameters of longistatin and compared them with those of t-PA. Longistatin was shown to bind with fibrin with the estimated K_d_, B_max_ and molar binding ratio (MBR) of 145.5±3.3 nmol/L, 3.1±0.6 µmol/L and 42.3±7.4, whereas those of t-PA were 159.2±7.4 nmol/L, 1.4±0.4 µmol/L and 19.3±4.7, respectively (Table 1), suggesting that longistatin potently binds with fibrin. Fibrin binding is an essential feature for the plasminogen activators that specifically activate fibrin clot-bound plasminogen. For example, t-PA is a very weak activator of plasminogen in the absence of fibrin. The affinity between t-PA and plasminogen is significantly increased in the presence of fibrin of blood clot [6], [33]. Fibrin binding was also assumed as critical in the activation process of plasminogen by longistatin.

**Table 1:** Comparison of fibrin-binding parameters of longistatin with those of t-PA.

Protein	K_d_ (nmol/L)	B_max_ (µmol/L)	MBR^a^
Longistatin	145.5±3.3	3.1±0.6	42.3±7.4
t-PA	159.2±7.4	1.4±0.4	19.3±4.7
	p<0.05	p<0.01	p<0.01

a Expressed as moles of longistatin/t-PA per mole of fibrin. Determined at maximum binding.

### Longistatin activates plasminogen present in plasma milieu and induces thrombolysis

**Figure 7:**
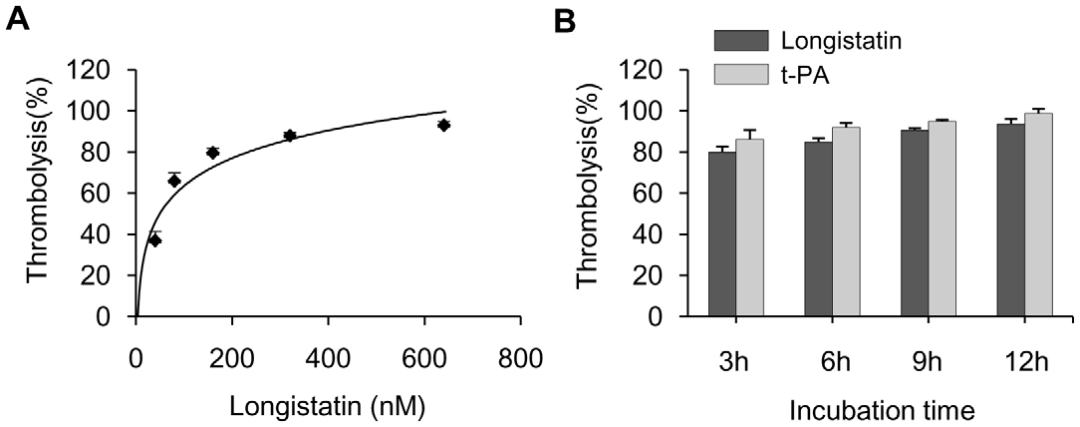
Lysis of platelet-rich thrombi by longistatin in dog plasma. (A) Longistatin hydrolyzes platelet-rich thrombi *ex vivo* in dog plasma. Platelet-rich clot was produced by incubating 0.2 ml of dog blood and the thrombi were treated with longistatin at various concentrations in 0.5 ml of dog plasma at 37°C for 12 h and weighed at the indicated period. (B) Comparison of thrombolytic activity of longistatin with that of t-PA. Thrombi were treated with longistatin (640 nM)/t-PA (154 nM) under the same *ex vivo* experimental conditions.

To demonstrate the thrombolytic capability of longistatin, we treated freshly prepared platelet-rich thrombi kept in fresh plasma. Longistatin was able to cause lysis of platelet-rich thrombi in the presence of fresh plasma in a concentration-dependent manner and efficiently lysed thrombi at 640 nM concentration (Figure 7A). Longistatin induced more than 50% lysis of thrombi within 2 h at 640 nM concentration (data not shown). In the same experimental setup, longistatin (640 nM) and t-PA (154 nm) induced 93.62%±2.33% and 98.78%±2.11% lysis of thrombi, respectively, by 12 h (Figure 7B). Moreover, like t-PA, longistatin efficiently digested fibrin clot produced from purified, commercially available fibrinogen and thrombin in the presence of plasma (data not shown). Taken together, our results suggest that longistatin is capable of causing thrombolysis and subsequent recanalization of occluded thrombosed vascular tree by activating the physiological level of plasminogen into plasmin.

### Functional implications of longistatin in blood coagulation and fibrinolysis cascades

**Figure 8:**
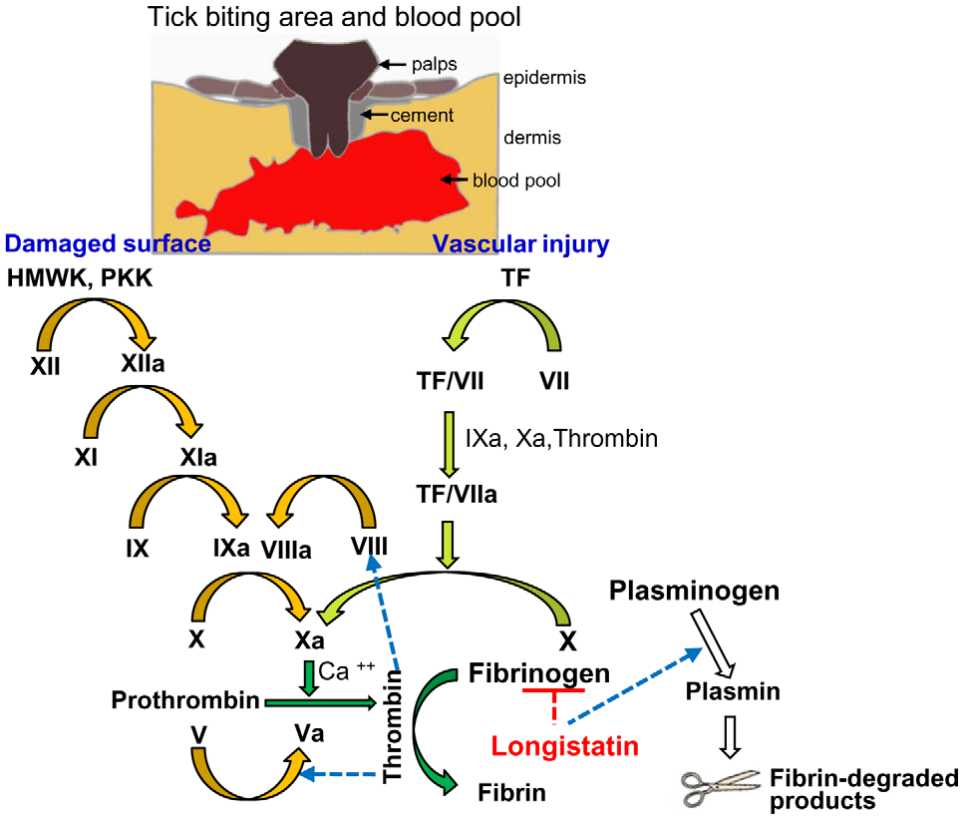
A schematic diagram showing roles of longistatin in blood coagulation and fibrinolysis events. In the initial phase, the tick bites and lacerates tissues at the site of attachment and damages vascular beds, which results in hemorrhage leading to the development of a blood pool. Longistatin is synthesized in and secreted from the salivary glands and injected into the blood pool during feeding process [27]. Longistatin degrades fibrinogen and activates plasminogen to its active form, plasmin. HMWK, high-molecular-weight kininogen; PKK, prekallikrein; TF, tissue factor. Yellow arrows, contact activation (intrinsic) pathway; olive-green arrows, tissue factor (extrinsic) pathway; green arrows, common pathway of coagulation cascade and white arrows, fibrinolytic pathway. Figure adapted from ref. [1], [6], [32].

We hypothesized that ixodid ticks synthesize longistatin and possibly other functionally related bioactive molecules to efficiently counteract host's hemostatic ability and/or to activate its own fibrinolytic machinery to create blood pools for the acquisition of blood-meals and engorgement. Our *in vivo* and *in vitro* data strongly support this hypothesis and that longistatin exerts its multifunctional roles both in coagulation cascades and in fibrinolytic pathways. In this study, we proposed a longistatin-induced anti-coagulation and fibrinolytic mechanism that works persistently against host's hemostatic pathways until ticks ensure full blood-meals (Figure 8).

## Discussion

Previous literatures on the feeding behavior and physiology of hematophagous ixodid ticks suggest that acquisition of blood-meals from mammalian hosts is modulated by a vast array of pharmacologically active bio-molecules secreted from the salivary glands of these amazing tiny arthropods. Ticks' saliva is thought to efficiently manipulate the hosts' strong defense mechanisms, such as hemostasis, inflammatory reactions and immune responses that are induced against the tick during hematophagy [1], [26], [34]–[37]. In the present study, we provide evidence that longistatin, a salivary gland protein with two functional EF-hand Ca^++^-binding domains isolated from the ixodid tick, *H. longicornis*, binds with fibrin and specifically activates plasminogen to its active form, plasmin. We also demonstrate that longistatin degrades fibrinogen and is able to delay fibrin clot formation; thus, longistatin appears to be essential to keep the blood in a fluid state in the blood pool, which enables ticks to feed and replete on blood-meals from hosts.

Our *in vivo* gene silencing study revealed that longistatin-specific gene-knockdown ticks were unable to produce a blood pool and consequently failed to replete. These findings prompted us to unveil the underlying complex mechanism(s) by which longistatin exerts its modulatory roles in blood pool formation and maintenance, which is an essential pre-requisite to ticks for successful feeding and engorgement. Anticoagulation of host's blood is a crucial step for the development and maintenance of a blood pool. To verify this, we have conducted several anticoagulation and fibrinolysis assays. Interestingly, we have shown that longistatin significantly extends the time of fibrin clot formation up to 90 min when fibrinogen is treated with longistatin compared with untreated fibrinogen (control), which takes only 15 min to complete fibrin clot formation. Data on fibrinogenolytic assays also support this observation that longistatin is capable of degrading the α, β and γ chains of fibrinogen in a concentration-dependent manner and completely hydrolyzes these three bands. Therefore, it may be assumed that the delay in the fibrin clot formation during *in vitro* anticoagulation assays in the presence of longistatin is due to the gradual degradation of coagulable proteins in the reaction mixture. Fibrinogenolytic activity has also been reported in tick metalloprotease isolated from *Ixodes scapularis* [38] and in several proteases identified from snake venoms [39] and spider toxins [40]. Fibrinogen is the key component for the formation of cross-linked fibrin polymer. In general, after laceration of blood vessels, platelets are exposed to the subendothelial collagen and become activated and aggregated around the injuries. Then, fibrin strands, derived from cleavage of fibrinogen, intertwine about the aggregated platelets giving rigidity and stability of the initial and preliminary platelet plugs. Finally, activated factor XIII forms covalent bonds that crosslink the fibrin polymers and thus the injured blood vessels are sealed by an extra strengthened, stable fibrin clot leading to hemostasis [1], [41], [42]. Therefore, it may be predicted that fibrinogenase activity of longistatin hampers hemostasis and may facilitate hemorrhage into the blood pools from which ticks persistently feed blood-meals.

We have shown that longistatin specifically activated plasminogen in the presence of fibrin clot and cleaved it into plasmin, heavy chain (α) and light chain (β). Although plasminogen is unable to cleave fibrin clot but it has a strong affinity for fibrin. Plasminogen has a secondary structure known as a kringle domain, which anchors plasminogen specifically to the carboxy-terminal arginine and lysine residues of the fibrin; thus, plasminogen becomes more concentrated on the surface of the clot, whenever and wherever it develops. As soon as plasminogen is converted into plasmin by its activators, it functions like a serine protease. Fibrin acts as a cofactor in the enzymatic activation reactions when plasminogen is activated by the fibrin-selective agents, like t-PA and its derivatives or staphylokinase and its derivatives. Through a highly orchestrated biochemical process, plasmin initially creates nicks on the fibrin and further digestion leads to the complete dissolution of fibrin meshwork into soluble fibrin degradation products [6], [32], [43]. Our results clearly demonstrate that longistatin contributes to this well-coordinated fibrinolytic pathway by activating plasminogen into plasmin. Plasminogen activators have been purified from the venomous snake, *Trimeresurus stejnegeri* [44] and from the saliva of common vampire bat, *Desmodus rotundus* [10], [45]. In vampire bats, plasminogen activator is thought to be a key enzyme that plays a crucial role in the maintenance of the flow of blood during the feeding process [46] and snake-venom plasminogen activator is associated with the pathogenesis of envenomation rendering the blood incoagulable [44]. Maintenance of hosts' blood fluidity at the site of biting is also very critical for the development of blood pool and subsequent blood feeding, and eventually for the survival of ixodid ticks. From the available evidence, here we assume that fibrin-specific, plasminogen-activation-dependent thrombolytic potentiality of longistatin plays significant functional roles in the pathobiology of vector ticks through the formation of feeding lesion and by the maintenance of its homeostasis throughout the entire period of blood feeding.

In conclusion, longistatin, a salivary-gland protein of ixodid ticks with multitarget potential, shows high specificity for fibrin clot-bound plasminogen, and degrades fibrinogen. These findings indicate that longistatin plays significant roles in the formation and maintenance of blood pools leading to the successful acquisition of blood-meals and may be critical for the survival of ixodid ticks. Furthermore, our data suggest that longistatin may serve as a novel therapeutic target against ticks and tick-borne diseases, including human diseases such as thrombosis or other occlusive cardiovascular accidents.

## Materials and Methods

### Ticks and animals

We propagated parthenogenetic Okayama strains of *H. longicornis* at the Laboratory of Parasitic Diseases, National Institute of Animal Health (NIAH), Tsukuba, Japan, by feeding on the ear of tick-naïve, specific-pathogen-free Japanese White rabbits according to methods described previously [27]. Ticks were collected after detachment following full engorgement or at the indicated period of feeding. After collection, ticks were weighed and phenotypic differences between the ticks of RNAi and control groups were recorded. This study was carried out in strict accordance with the recommendations in the Guide for the Care and Use of Laboratory Animals of the National Institutes of Animal Health. The protocol was approved by the Committee of the Ethics of Animal Experiments of the NIAH (Permit Number: 09-017, 09-018, 10-008, 10-010). All surgeries were performed under sodium pentobarbital anesthesia, and all efforts were made to minimize the animals' suffering.

### Reagents

Green fluorescent-labeled secondary antibody (Alexa Flour 488 goat anti-mouse IgG (H+L)) was purchased from Invitrogen and alkaline phosphatase-conjugated goat anti-mouse IgG (H+L) was from ZYMED. Nitroblue tetrazolium/5-bromo-4-chloro-3-indolyl phosphate (BCIP/NBT) and T7 RNA polymerase were from Promega. Purified human plasmin, bovine thrombin and fibrinogen were obtained from Sigma. Purified human plasminogen, t-PA and anti-t-PA (Ab-1) mouse monoclonal antibody (GMA-043) were purchased from Calbiochem. Human u-PA was from Cosmo Bio Co. LTD and polyclonal antibody to u-PA was from Acris Antibodies GmbH. Fibrin CNBr fragment was obtained from Technoclone and Boc-Glu-Lys-Lys-MCA from Peptide Institute. Total RNA extraction kit and DNA gel extraction kit were purchased from QIAGEN.

### RNA interference

We performed RNAi using dsRNA following a protocol described previously [47]. The sequence coding longistatin was cloned into pBluescript II SK^+^ vector in *Xho*I and *Eco*RI restriction sites following a protocol described previously [27]. The dsRNA complementary to the *E. coli mal*E gene that encodes maltose-binding protein was used as a negative control. cDNA corresponding to *mal*E mRNA was synthesized and was cloned into pBluescript II SK^+^ plasmid using the primers 5′-CCGCTCGAGCGGTTATGAAAATAAAAACAGGTGCA-3′ and 5′-GAATTCGCTTGTCCTGGAACGCTTTGTC-3′. The inserted sequences of longistatin and *mal*E were amplified by PCR using primers T7 (5′-GTAATACGACTCACTATAGGGC-3′) and CMO422 (5′-GCGTAATACGACTCACTATAGGGAACAAAAGCTGGAGCT-3′) to attach to T7 promoter recognition sites at either end. The PCR products were purified using gel extraction kit (QIAGEN). dsRNA complementary to the respective DNA inserts was synthesized by *in vitro* transcription using T7 RNA polymerase (Promega). One microgram of longistatin dsRNA (ds*longistatin*, treated group) or *mal*E dsRNA (ds*mal*E, control group) was injected into each tick through the 4^th^ coxa. Ticks were observed in a humified incubator for 24 h at 25°C prior to attaching them on the host for feeding. A total of 120 ticks, each of 60 in RNAi and control groups, were attached on the ear of tick-naïve rabbits. Ticks were collected at 24, 48, 72 and 96 h of feeding or after repletion. All ticks collected after they had dropped off the host following full engorgement were weighed individually using a digital balance (Sartorius).

### Semiquantitative RT-PCR and qRT-PCR

Salivary glands from adult ticks of both control and RNAi groups at different feeding periods (24, 48, 72, 96 h and engorged) were collected as described previously [27]. Shortly after collection, salivary glands were submerged in RNA*later*, an RNA Stabilization Reagent (QIAGEN). Total RNA was isolated using an RNeasy Mini Kit (QIAGEN) according to the manufacturer's protocol and 500 ng of total RNA was used for reverse transcription before PCR. Single-stranded cDNA was prepared using Takara RNA PCR Kit (AMV) Ver.3.0 (Takara) following the manufacturer's instructions. A series of PCRs were carried out using 500 ng of cDNA from each sample and longistatin-specific oligonucleotides (5′GCTATCTCGGCTCCTGTGTC 3′ and 5′ATCTTCGCCAGGTCCTTCTT 3′) or oligonucleotides specific for a control cDNA encoding β-actin in a final volume of 20 µl. The PCR product was subjected to electrophoresis in 1% agarose gel. The qRT-PCR was performed in a LightCycler 1.5 instrument (Roche Instrument Centre AG) using LightCycler FastStart DNA Master SYBR Green I (Roche Diagnostics) following a procedure described previously [48]. The reaction mixture of 20 µl contained 4 mM MgCl_2_, 0.5 µM of each primer (forward and reverse as described above) and 2 µl (250 ng/µl) of the single-stranded DNA template. The data obtained were analyzed using LightCycler Software Version 3.5.

### Recombinant longistatin and polyclonal antibody production

Recombinant longistatin was produced, purified, dialyzed and its concentration was determined as previously described [27]. Purified longistatin was kept at –20°C until further use. Polyclonal antibody was produced in BALB/c mice following a procedure described elsewhere [27].

### Immunofluorescence and histopathology

We collected salivary glands from partially fed (96 h) adult ticks of both control and RNAi groups as described above. Salivary glands were placed on slides and were fixed with 4% paraformaldehyde. Salivary glands were then permeabilized with 0.1% tritonX-100 and were treated with mouse anti-longistatin sera (1∶100). Bound antibodies were detected using green fluorescent-labeled secondary antibody (Alexa Flour 488 goat anti-mouse IgG (H+L), Invitrogen). Slides were mounted with VECTASHIELD mounting medium containing 4′,6-diamidino-2-phenylindole (DAPI, Vector Laboratories) and examined under a fluorescent microscope (Leica). In addition, thin sections were prepared from tissues collected from the feeding lesions developed on a rabbit's ear at the site of attachment of the ticks. Tissue sections were then subjected to immunofluorescent staining using mouse anti-longistatin sera as described previously[27]. Rabbit's tissues were also stained with EVGS as described previously [49].

### Immunoblot analysis

Equal numbers of adult ticks from both RNAi and control groups collected at different feeding intervals (24, 48, 72, 96 h and engorged) were dissected separately in PBS and salivary glands were isolated. Antigens were prepared as previously described [50]. Equal amounts of protein (4 µg) were separated by 12.5% SDS−PAGE under reducing conditions and were transferred onto nitrocellulose membrane. The membrane was treated with mouse anti-longistatin sera (1∶100) overnight at 4°C. Bound antibodies were probed with alkaline phosphatase-conjugated goat anti-mouse IgG (H+L) (ZYMED). The membranes were developed with nitroblue tetrazolium/5-bromo-4-chloro-3-indolyl phosphate (BCIP/NBT, Promega).

### Anticoagulation and fibrinogenolytic assays

Fibrinogen (7.5 mM) was pre-incubated in a total volume of 397 µl of buffer containing 25 mM Hepes (pH 7.2) and 25 mM NaCl in the absence or presence of longistatin (0.1, 0.2, 0.4, 0.8 and 1.6 µM) or plasmin (1.6 µM) at 25°C for 3 h. Fibrin clot formation was initiated by the addition of 3 µl of thrombin (0.10 NIH unit/µl). Fibrin clot formation was detected visually and also by determining changes in turbidity at OD_450_ using a spectrophotometer (Beckman Coulter) at 15 min intervals. To detect the fibrinogenolytic activity of longistatin, fibrinogen (7.5 mM) was incubated in a total volume of 100 µl of buffer (25 mM Hepes, pH 7.2, and 25 mM NaCl) in the absence or presence of longistatin (0.4, 0.8 and 1.6 µM) or plasmin (1.6 µM) at 25°C for 48 h. Aliquots were collected and separated by 12.5% SDS–PAGE.

### Plasminogen activation assays

Longistatin at various concentrations (40, 80, 160, 320 and 640 nM) was incubated in a 96-well cell culture plate without or with plasminogen (0.24 units) in the absence or presence of fibrin CNBr fragments (0.25, 1 and 4 µg) in a total volume of 200 µl of buffer (50 mM Tris–HCl, pH 7.5; 100 mM NaCl and 5 mM CaCl_2_) at 25°C for 2 h. Then, 2 µl (100 µM, final concentration) of plasmin-specific fluorogenic substrate (Boc-Glu-Lys-Lys-MCA) was added. Substrate hydrolysis was monitored by measuring excitation and emission wavelengths of 360 nm and 460 nm, respectively, at 15 min intervals using a Spectra Fluor fluorometer (TECAN, Männedorf, Switzerland). All assays were performed in triplicate and activity of activated plasminogen was expressed in an arbitrary fluorescent unit per min (AFU/min).

### Fibrinolytic assays

Fibrin clot was incubated in the presence or absence of longistatin. An initial fibrin clot was produced by incubating 10 µl of fibrinogen (7.5 mM) and 5 µl of thrombin (0.10 NIH unit/µl) in a total volume of 400 µl of buffer (50 mM Tris–HCl, pH 7.5; 100 mM NaCl and 5 mM CaCl_2_) at 25°C for 1 h. An equal volume of plasminogen (2.4 units), t-PA (154 nM) or longistatin at various concentrations (40, 80, 160, 320 and 640 nM) in a total volume of 100 µl of buffer, as mentioned above, was added to the fibrin clot and incubated at 25°C for 24 h. Lysis of fibrin clot was detected visually and also by measuring changes in turbidity at OD_450_ using a spectrophotometer (Beckman Coulter) at different time intervals (0, 6, 12 and 24 h). Furthermore, to verify the plasminogen activation potential of longistatin, 20 µl of digested products of clots corresponding to each concentration were collected and subjected to 12.5% SDS–PAGE analysis under reducing conditions.

### Fibrin clot binding assays

Fibrin binding assays were conducted with the modifications of procedures described previously [31]. Briefly, purified fibrinogen (3.75, 7.5, 15, 30 and 60 mM; final concentration) was mixed in the absence or presence of longistatin (10 µg) or an equal amount of t-PA or u-PA in a total volume of 200 µl of buffer (50 mM Tris–HCl, pH 7.5; 100 mM NaCl and 5 mM CaCl_2_). Fibrin clot formation was initiated by adding 3 µl of thrombin (0.10 NIH unit/µl) immediately and was incubated at 25°C for 1 h. The clot was centrifuged at 10,000 *g* for 10 min and supernatant was collected. The remaining clot was extensively washed in PBS and treated with anti-longistatin (1∶100), anti-t-PA (1∶100), anti-u-PA (1∶20) or pre-immune sera (1∶100) overnight at 4°C. Bound antibodies were detected using green fluorescent-labeled secondary antibody. Supernatant was analyzed by 12.5% SDS−PAGE under reducing conditions. The target protein band was excised and protein was extracted from the gel using negative zinc staining kit following the manufacturer's instructions (Bio-Rad). Double volume of cold (−20°C) acetone was mixed thoroughly with the extracted protein, incubated at −20°C for 1 h and centrifuged at 10,000 *g* for 30 min. The pellet was air-dried and dissolved with distilled water. The concentration of the extracted protein was determined using micro-BCA reagents (Pierce). To determine the binding parameters, longistatin (2−10 µg) or an equal amount of t-PA was incorporated into the fibrin clot. Residual longistatin/t-PA was extracted from the supernatant and the concentration of the relevant protein was determined following the same methods as mentioned above. K_d_, B_max_ and MBR were calculated according to the procedures as previously described [51].

### *Ex vivo* thrombolysis assays

Platelet-rich thrombi were produced by incubating 0.2 ml of fresh dog blood in a 96-well flat-bottom cell culture plate for 15 min. Thrombi were removed and washed gently with normal saline and weighed using a digital balance (Sartorius). The thrombi were then treated with t-PA (154 nM) or longistatin at various concentrations (40, 80, 160, 320 and 640 nM) in a total volume of 0.5 ml of fresh dog plasma at 37°C for 12 h and weighed at 3 h intervals. Furthermore, fibrin clot was produced by incubating purified, commercially available fibrinogen (7.5 mM) and thrombin (0.10 NIH unit/µl) as mentioned above and was treated with t-PA (154 nM) or longistatin (640 nM) in fresh dog plasma following the same experimental procedures and conditions.

### Accession numbers of the proteins and the genes used

Plasmin from human plasma (**NP_000292**), plasminogen from human plasma (**AAN85555**), t-PA from human plasma (**NP_000921**), u-PA from human plasma (**NP_002649.1**), thrombin (as prothrombin) from bovine plasma (**NP_776302**), fibrinogen from bovine plasma (α chain, **AAI02565**; β chain, **NP_001136389** and γ chain, **AAI02630**), longistatin from *H. longicornis* (**AB519820**) and *mal*E from *E. coli* (**ZP_02999303**).

### Statistical analysis

Data were presented as mean ± standard error, where appropriate. Statistical significance was determined using Student's *t* test with unequal variance.
